# Generation of Nano-Bubbles by NaHCO_3_ for Improving the FO Membrane Performance

**DOI:** 10.3390/membranes13040404

**Published:** 2023-04-03

**Authors:** Shilin Zhou, Yuzhe Zhou, Jin He, Yuwen Lai, Yanchun Li, Wentao Yan, Yong Zhou, Congjie Gao

**Affiliations:** Center for Membrane Separation and Water Science & Technology College of Chemical Engineering, Zhejiang University of Technology Hangzhou, Hangzhou 310014, China

**Keywords:** thin-film composite membrane, polarization concentration, forward osmosis, micro-nano structure

## Abstract

Thin-film composite (TFC) polyamide membranes have a wide range of applications in forward osmosis, but tuning the water flux remains a significant challenge due to concentration polarization. The generation of nano-sized voids within the polyamide rejection layer can change the roughness of the membrane. In this experiment, the micro-nano structure of the PA rejection layer was adjusted by adding sodium bicarbonate to the aqueous phase to generate nano-bubbles, and the changes of its roughness with the addition of sodium bicarbonate were systematically demonstrated. With the enhanced nano-bubbles, more and more blade-like and band-like features appeared on the PA layer, which could effectively reduce the reverse solute flux of the PA layer and improve the salt rejection of the FO membrane. The increase in roughness raised the area of the membrane surface, which led to a larger area for concentration polarization and reduced the water flux. This experiment demonstrated the variation of roughness and water flux, providing an effective idea for the preparation of high-performance FO membranes.

## 1. Introduction

Desalination and water treatment have been widely used with thin-film composite forward osmosis (TFC-FO) membranes. The forward-osmosis filtration process is becoming more and more popular in desalination and water treatment because of its low energy consumption [1,2]. Research on FO membranes has been increasing in recent years. There are two main types of forward osmosis membranes used in the FO process. One is the integrated asymmetric membrane prepared by the phase conversion method [3,4,5], which allows separation by a denser skin layer formed on the membrane surface [6]. The integrated asymmetric membrane has some drawbacks, such as a narrow range of applicable pH, a low temperature tolerance, and limited applications in wastewater and sewage treatment. The other type is the thin-film composite (TFC) membrane [7,8,9], which consists of a porous support layer for mechanical support and a dense selective layer for selective separation, usually prepared by interfacial polymerization (IP). The advantage of composite membranes over integrated asymmetric membranes is that the selective and support layer can be optimized separately and has great potential for handling different types of feed solutions [10,11,12]. On the basis of the desired performance, it is possible to develop high-performance FO membranes that meet the necessary requirements according to the characteristics of the FO application. TFC membranes are based on polyamide chemistry [13,14] and are prepared by the IP reaction of amine m-phenylenediamine (MPD) and tri-mesoyl chloride (TMC) that forms the PA layer, which is a thin salt rejection layer. Some early studies reported the (apparent) thickness of the rejection layers in the range of 50–300 nm [15,16]. Thin-film composite membranes exhibit nanoscale non-uniform surfaces with unique roughness features, often referred to as “ridge and valley” structures [17]. The separation performance of TFC-FO membranes is critically related to the surface roughness of the polyamide layer.

The formation of rough features in PA layers is usually explained by the instability of the reaction interface when amine monomers MPD diffuse into organic solutions of chlorides TMC [16,17,18]. However, these simple models cannot explain the disappearance of the “ridge and valley” structure at low MPD–TMC concentrations [17,19,20]. In a recent study, Ma et al., reported that the generation of acid and heat during the IP reaction, resulting in the release of nano-bubbles of dissolved gases (e.g., CO_2_) from ammonia, is responsible for the formation of rough features in the PA layer [17,20,21]. The presence of a large number of nano-sized voids (up to 32% volume fraction) in the polyamide layer supports this nano-foaming mechanism to some extent [22]. A series of methods further explained the disappearance of surface roughness by pretreatment with aqueous and oil-phase solutions or by modulation of the IP process, through the use of low TMC and MPD concentrations [19,23] and the pre-degassing of the amine solutions before IP [21] or through the prolongation of the reaction time by electrospray-assisted IP [21,24]. A significant problem in the FO membrane is the internal concentration polarization (ICP) effect, which is mainly caused by the formation of concentration gradients in the porous support layer [25]. However, the impact of nano foaming on the surface roughness alone has rarely been considered for FO membranes, for example, in terms of how roughness variations may impact concentration polarization.

The study of Ma [21] demonstrated that nanopores are formed due to the release of nano-sized bubbles during IP, and the fine bubbles released in the case of interfacial polymerization are encapsulated and lead to the appearance of roughness features. It was found that the water flux gradually decreased as the nano-bubbles promoted the increase in roughness. Based on the effect of the forward osmosis membrane concentration polarization, the larger the membrane roughness, the larger its structural parameters, and maybe, the larger the area where concentration polarization occurs. Thus, the flux is gradually decreased. In order to investigate the effect of nano-bubbles generation on forward osmosis membrane flux and reverse salt flux, a series of concentration gradients of sodium bicarbonate solutions were designed to induce more nano-bubbles and thus increase the membrane roughness so to investigate the effect on water flux variation. We further demonstrated enhanced nanobubble formation and the PA layer’s morphology and separation properties. Our study provides insight into the basic mechanism of polyamide membrane formation and contributes a new idea to tune the PA layer structure and separation performance.

## 2. Materials and Methods

### 2.1. Materials

All reagents, including sodium bicarbonate (NaHCO_3_, powder, AR), m-phenylenediamine (MPD, 99%, flakes), tri-mesoyl chloride (TMC, 98%), n-hexane (AR), N, N-dimethylformamide (DMF, ≥99.9%), sodium chloride (anhydrous NaCl, AR) were purchased from Sigma-Aldrich (St. Louis, MO, USA) and were not purified. Polysulfone (PSF) ultrafiltration membranes from Zhejiang University of Technology Huzhou Research Institute (Huzhou, China) were used.

### 2.2. Membrane Preparation

In this experiment, aromatic polyamide composite forward-osmosis (TFC FO) membranes were prepared by the IP method. The aqueous phase solution was prepared by adding 2% (*w*/*v*) MPD and then NaHCO_3_ at the concentration of 0, 2, 4, 6, and 8 wt%, followed by stirring the solution for 2 h to obtain a mixed homogeneous aqueous-phase solution. Then, different concentrations of sodium bicarbonate solution were added under magnetic stirring to obtain complete dissolution. The oil phase solution was prepared by adding 0.05% (*w*/*v*) TMC to a hexane solution under magnetic stirring for 3 h. The PSF base film was first soaked in deionized water for 12 h as a support layer and rinsed with pure water two to three times before use.

The process of interfacial polymerization was as follows: first, the membrane surface was impregnated with the configured aqueous-phase solution for 2 min, then the excess water on the PSF membrane surface was eliminated with filter paper, and finally the visible water droplets were blown off with an ear wash ball. Next, the PSF membrane surface was impregnated with the oil phase, and interfacial polymerization occurred at the interface between the water phase and the oil phase. Then, 30 s later, the oil-phase solution was poured out, and the membrane was immediately rinsed twice with the hexane solution to wash away the residual solvent. Finally, it was placed into an oven for post-treatment at 80 °C for 10 min. The produced TFC membranes were TFC-0, TFC-2, TFC-4, TFC-6, and TFC-8. The finished membranes were washed with pure water and stored in sterile water before the next procedures.

### 2.3. Membranes Characterization

A field-emission scanning electron microscope was used in this experiment to observe the morphological changes on the surface, backside, and cross-section of the TFC film at an operating voltage of 10 kV. Atomic microscope analysis images and membrane surface roughness were analyzed by atomic force microscopy (AFM, Dimension Icon, Bruker, Ettlingen, Germany). The membrane surface zeta potential charged property is one of the critical properties of membranes and has a massive impact on a membrane’s separation performance and contamination resistance. The membrane surface zeta potential was examined using a solid-surface zeta potential tester (SurPASS 3, Anton Paar, Graz, Austria) in the pH range of 3–10, with a test solution of 0.01 mmol/L KCl and titrated pH with a 0.05 mol/L NaOH solution and a 0.05 mol/L hydrochloric acid solution; the samples size was 2 cm × 1 cm × 2 pieces.

### 2.4. Filtration Experiments

**Figure 1:**
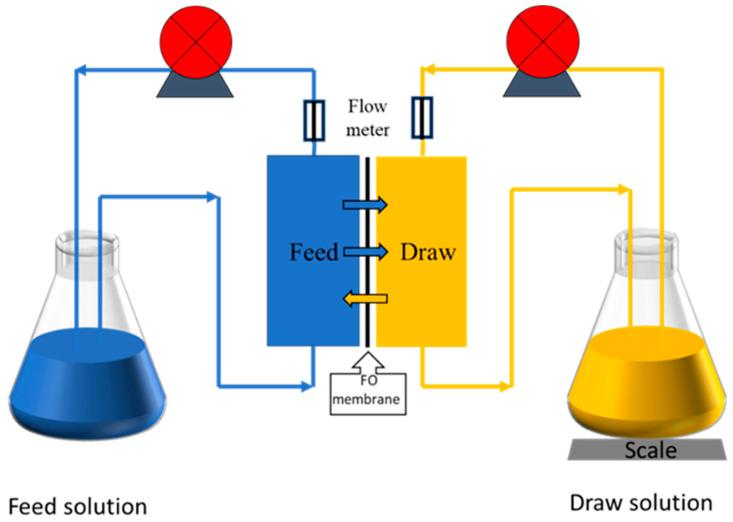
Schematic diagram of the FO experimental process.

The FO performance test using a homemade FO device is shown in Figure 1. Deionized water was used as the feed solution [26], involving the draw solution NaCl, with a concentration of 1 M. The effective membrane area of the membrane cell was 40 cm^2^. During the test, the circulation flow rate of both the draw solution and the feed solution was kept at 0.15 L/min, and the temperature was maintained at about 25 °C. According to the orientation of the FO membrane, this experiment was tested in the FO mode (with the selected surface facing the feed solution), and the water flux Jv (L·m^−2^·h^−1^) and the reverse salt flux Js (g·m^−2^·h^−1^) could be calculated according to Equations (1) and (2):

(1) $$Jv=\frac{\Delta m}{Am\Delta t\rho }$$

(2) $$Js=\frac{\Delta (CtVt)}{Am\Delta t}$$

In Equation (1), Δ*m* is the changing mass on the side of the feed solution at the interval time ∆*t*, measured by an electronic balance, *Am* is the effective membrane area, and *ρ* (kg·L^−1^) is the density of deionized water. In Equation (2), *Ct* and *Vt* are the concentration and volume of the raw material liquid at time *t*, respectively; the concentration of the raw material liquid was measured by a conductivity meter and converted according to the linear variation relationship between conductivity and concentration. The structural parameter *S* was measured by Equation (3):

(3) $$Jv=D/S\left[Ln\frac{A\pi_{draw}+B}{A\pi_{feed}+B+Jv}\right]$$

where *D* is the solute diffusion coefficient, and *π_draw_* and *π_feed_* are the osmotic pressure of the extracted solution and the feed solution, respectively. Therefore, the structural parameter *S* could be obtained from the equation where *A* is the water permeability coefficient, and *B* is the salt permeability coefficient, using a reverse osmosis device to measure the *A* and *B* values, and the final separation performance of the membrane was measured by a staggered-flow RO filtration device with the following feed solution and measurement conditions: (i) pure water flux measurement, where the feed was deionized water at a pressure of 1.0 MPa, and the flow rate was controlled at about 3 L·min^−1^; (ii) 1000 ppm water retention measurement, where the feed was a 1000 ppm NaCl solution, the pressure was 1.0 MPa, and the flow rate was controlled at 3 L·min^−1^. The diameter of the circular membrane cell was 5 cm, the tank depth of the feed solution was 2.5 mm, and the effective membrane cell area was 21.23 cm^2^. The permeate was recovered for volume and conductivity measurements. Three parallel samples were taken for each membrane, and the results were averaged. The formula used is

(4) $$A=\frac{J_{w}}{∆P}$$

In Equation (4), $J_{w}$ (L·m^−2^·h^−1^) is the pure water flow rate, ∆*P* (bar) is the test pressure, *A* (L·m^−2^·h^−1^·bar^−1^) is the water permeability coefficient through the polyamide membrane.

(5) $$J=\frac{∆V}{S\cdot ∆t}$$

In Equation (5), ∆*V* (L) is the infiltration volume, *S* (m^2^) is defined as the infiltration area over which water flows, ∆*t* is the test time, and *J* (L·m^−2^·h^−1^) is the infiltration flux.

(6) $$B=\frac{Js}{C_{f}-C_{p}}=J_{sw}\times \frac{1-R}{R}$$

In Equation (6), $J_{sw}$ (L·m^−2^·h^−1^) is the water flux, including the solvents DI water and solute NaCl, Js (L·m^−2^·h^−1^) refers to the solute NaCl flux, $C_{f}$ (mg/L) and $C_{p}$ (mg/L) are the feed solution and the permeating solute NaCl concentration, respectively, *B* (L·m^−2^·h^−1^·bar^−1^) is the permeation coefficient of solute NaCl.

(7) $$R=\frac{C_{f}-C_{p}}{C_{f}}\times 100\%$$

## 3. Results and Discussion

### 3.1. Nanobubbles Regulate the Surface Structure

TFC-FO membranes prepared with 2.0 wt% MPD and 0.05 wt% TMC showed a relatively weak “ridge and valley” structure with an average roughness (Ra) of 60.45 nm, usually observed with polyamide FO membranes. In Ma’s [21] experiments, in order to verify that the roughness was produced by the release of dissolved gas, a set of m-phenylenediamine solutions were evacuated and then subjected to interfacial polymerization, and the roughness, measured by atomic force microscopy, appeared greatly reduced, proving to a certain extent that the release of dissolved gas produced nanopores, which allowed us to add carbon dioxide to the aqueous phase solution and thus generate heat and acid through the reaction to modulate the surface roughness. The addition of sodium bicarbonate to produce nano-bubbles was shown to have an impact on the IP process. The “ridges and valleys” on the surface of the PA film became more and more pronounced after the addition of sodium bicarbonate. This experiment demonstrated the systematic evolution of the rough structure of the polyamide film, with more and more leaf-like and band-like features appearing under enhanced nano-foaming conditions. The appearance of “ridges and valleys” resulted from trapped nano-bubbles in the polyamide. After forming a gas-tight polyamide film at the reaction interface, any further gas released had now to escape from its opposite side, interrupting the complete encapsulation of the nano-voids and resulting in the formation of honeycomb-like pores for air to pass through [27]. As the amount of sodium bicarbonate added increased, more and more honeycomb pores were created on the backside, indicating the production of more and more nano-bubbles, which affected the morphology of the PA layer, resulting in an increasingly pronounced “ridge and valley” structure.

**Figure 2:**
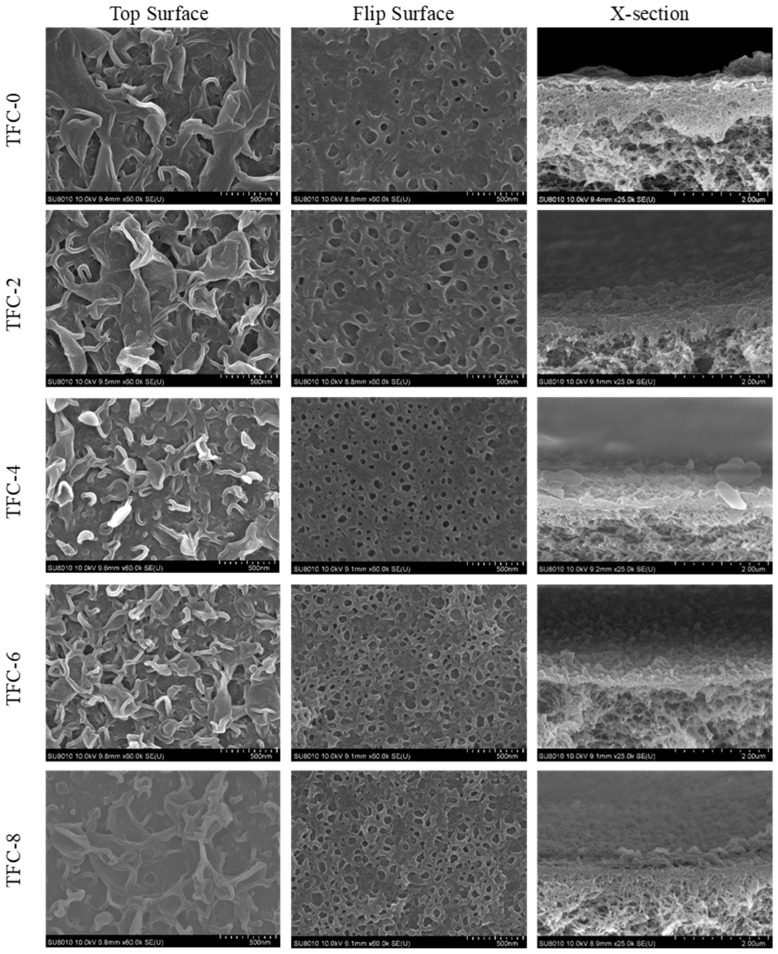
The top surface, flip surface, and cross-section SEM images of the FO membranes.

When the concentration of sodium bicarbonate was more than 6%wt, too much carbon dioxide was released, leading to the rupture of the polyamide layer. Because the reaction continued, the polyamide layer was broken by carbon dioxide, and then secondary interfacial polymerization occurred at its interface, because the polyamide layer created by the secondary interfacial polymerization was relatively gentle, and the roughness slowly decreased. Therefore, the roughness increased first and then decreased, and this trend could also be observed in atomic force microscopy and SEM cross-section images. By observing the TFC-0 “ridge and valley” structure the increasing roughness was not very obvious. The nodular feature of secondary interfacial polymerization continued to grow on leaf-like structure. When the concentration of sodium bicarbonate increased to 8%wt, the polyamide layer showed a volcanic eruption morphology, with the generation of nanobubbles and more defects, leading to more obvious cavities and secondary interfacial polymerization features, as shown in Figure 2.

The heat generated by the reaction reduced the solubility of dissolved gases (e.g., CO_2_, N_2_, and O_2_) and produced a strong acid (HCl) as a by-product. At the same time the added sodium bicarbonate further promoted CO_2_ degassing as the reaction proceeded. In the case of interfacial polymerization, the released nano-bubbles were encapsulated. As the sodium bicarbonate content increased, more and more CO_2_ was degassed, and the release of nano-bubbles made the film surface rougher and rougher, as shown in the plan view. The polyamide layer appeared to have a weaker “ridge and valley” structure when NaHCO_3_ was not added, as shown in the plan view of Figure 2, and the rough leaf-like features appeared to be flatter, probably due to the low mechanical strength of the rough features formed at low monomer concentrations. With the addition of sodium bicarbonate, which increased the formation rate of the nanobubbles, we obtained polyamide films with more leafy or nodular roughness.

**Figure 3:**
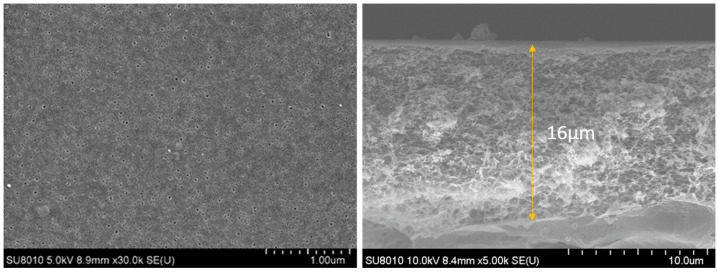
Surface pore size and PSF cross-section images of the substrate membrane.

By scanning electron microscopy of the backside of the PA layer, we found that the backside of the PA layer at all concentrations had honeycomb openings (as shown in Figure 2). The opening densities are shown in Table 1. The average opening diameters were in the range of 55–88 nm, and these data were in considerable agreement with previous studies [21,28,29]. We assumed that the backside openings were directly connected to the nanovoids in the PA layer [30,31]. The thickness of the PSF support layer was about 126 μm, and the pore size was 14.75 ± 0.21 nm, while the thickness of the polysulfone layer in the PSF support layer was about 16 μm. and the density of the PSF support layer pores was in the range of 349 ± 26/μm^2^. The images of the PSF support layer pores are shown in Figure 3. They appeared to be 3.7–15.9 times larger than the PA backside openings of the corresponding membranes, indicating that each opening could be connected to multiple pores in the substrate. With the addition of sodium bicarbonate, each opening connected fewer pores in the substrate. The increase of the pore size on the back side of the polyamide also proved the effect of nano-foaming on the polyamide layer. The gas in the nano-bubbles escaped from the pores of the substrate. Still, as the pore connection to the openings decreased, it was more difficult for the gas to escape from the openings and break through the polyamide layer to form a unique nodule and blade structure. When the concentration of sodium bicarbonate increased to a certain value, the openings of the substrate were too small to release the nano-bubbles generated during the reaction, and a secondary interfacial polymerization structure was created on the original structure, which reduced the roughness of its surface to a certain extent. This was also shown in previous studies, such as that of Song, where the effect of nano-bubbles on polyamide morphology was described in detail [27]. A feature similar to secondary interfacial polymerization appeared when the concentration of sodium bicarbonate was very high. The observed pattern was identical to those previously presented for polyamide surfaces [32].

### 3.2. Effect of Sodium Hydrogen Acid on the Roughness

**Figure 4:**
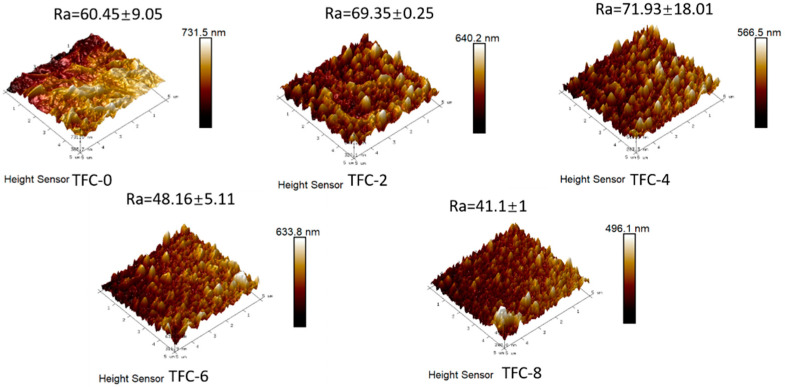
AFM surface roughness and surface area ratio of the TFC polyamide FO membranes.

The surface roughness of the TFC-FO membranes based on AFM measurements is shown in Figure 4. The roughness of the membranes prepared using MPD solutions without adding sodium bicarbonate were used as a negative control. The average roughness (Ra) values of the TFC-2 and TFC-4 membranes were much higher compared to that of TFC-0. It was found that the addition of sodium bicarbonate effectively increased the surface roughness of the polyamide membranes, which is also consistent with the characteristics of the polyamide layer surface observed by electron microscopy, as the addition of sodium bicarbonate produced more nodules and blade-like structures, leading to an increase in the surface roughness of the polyamide layer. However, as the concentration of sodium bicarbonate increased beyond 6 wt%, its roughness tended to decrease. This was related to the release rate of nanobubbles, which produced smoother secondary interfacial polymerization features in the polyamide layer when the sodium bicarbonate concentration increased, leading to a decrease in its roughness, as described in detail in the paper by Song [27] on the effect of nanobubbles on polyamide morphology. After the rupture of the rough structures (TFC-6, TFC-8), Ra decreased significantly because of the overall “inverted U-shaped” dependence of the AFM roughness on the nanobubbles [28].

### 3.3. Effect of Roughness on the Performance of FO Membranes

**Figure 5:**
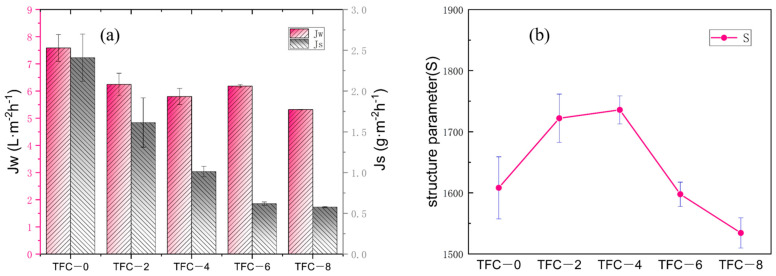
Water and salt transport properties of polyamide thin-film composite membranes: (**a**) membrane performance; (**b**) structure parameters S.

**Figure 6:**
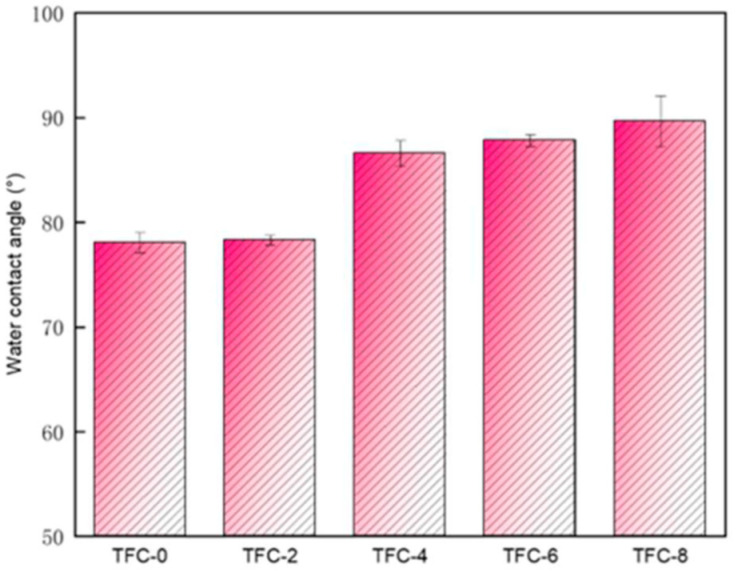
Water contact angle of MPD/TMC composite FO membranes with different concentrations of NaHCO_3_ in the aqueous phase.

In the process of adding sodium bicarbonate to adjust the membrane roughness, the surface roughness first increased and then decreased. We tested its water flux and reverse salt flux with homemade equipment (the device is shown in Figure 1). The changes of water flux and reverse salt flux are shown in Figure 5a. The water flux with the addition of sodium bicarbonate decreased compared to that observed with the blank film, before adding sodium bicarbonate at a concentration of 6 wt%. With the addition of sodium bicarbonate, the water flux became smaller and smaller. Since the membrane surface had a higher roughness after the addition of sodium bicarbonate, we assumed that when the roughness increased, the contact area of the membrane surface feed water was also larger, and the area with concentration polarization on the polyamide layer also increased [33,34]. This resulted in higher and higher structural parameters S (as shown in Figure 5b), and the water flux became smaller than that of TFC-0. At concentrations greater than or equal to 6 wt%, as the sodium bicarbonate concentration increased, the roughness showed a decreasing trend because, as the reaction proceeded, too much carbon dioxide was released, leading to the rupture of the polyamide layer. As the reaction proceeded, secondary structures, such as nodularity, appeared on the original leaves. This made the whole polyamide layer relatively smooth. When the concentration of sodium bicarbonate was greater than or equal to 6 wt%, secondary interfacial polymerization occurred, which reduced the roughness and increased the thickness of the film to a certain extent. The flux was relatively reduced, and when the concentration was greater than or equal to 6 wt%, the flux also tended to decrease. This is shown in Figure 6 that presents the water contact angle of MPD/TMC composite FO membranes with different concentrations of NaHCO_3_ added in the aqueous phase. We measured the contact angle on the surface of the polyamide layer, which showed an increasing trend, indicating that the membrane surface became more hydrophobic after the addition of sodium bicarbonate, and the hydrophilicity of the membrane surface affected the water flux of the membrane. Generally, less hydrophilicity leads to a lower water flux, which is consistent with the performance graph. This could be due to nanobubbles release, which rendered the membrane surface more hydrophobic.

**Figure 7:**
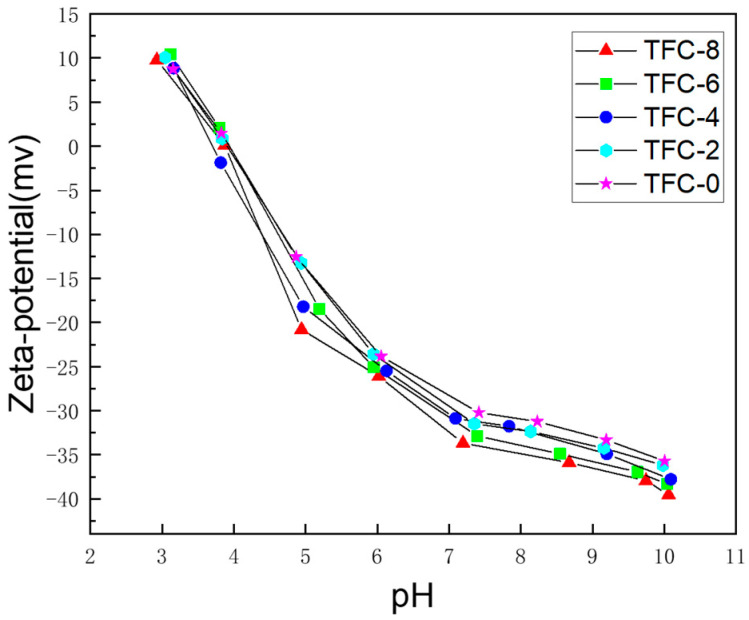
Zeta potential of MPD/TMC composite FO membranes with different concentrations of NaHCO_3_ in the aqueous phase.

**Figure 8:**
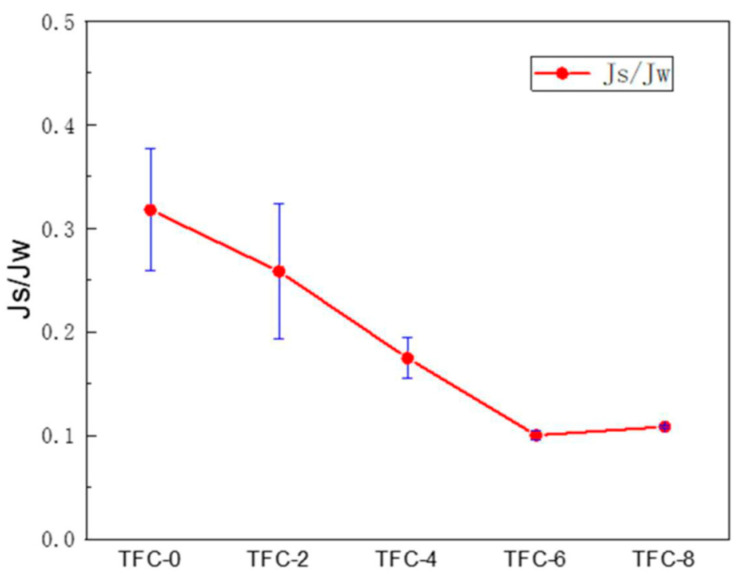
Js/Jw of MPD/TMC composite FO membranes with different concentrations of NaHCO_3_ in the aqueous phase.

The surface potential of TFC polyamide FO membranes based on zeta potential measurements is shown in Figure 7 where unreacted groups of amines and carboxylic acids resulting from the chloride hydrolysis of TMC molecules are visible on the membrane surface after interfacial polymerization. The potential increased with the addition of sodium bicarbonate, and its negative charge became larger at a pH between 7 and 10. The number of negative charges on the membrane increased accordingly. The reason for this phenomenon was the presence of more TMC molecules not undergoing hydrolysis as the amount of NaHCO_3_ added increased, due to the consumption of the hydrolysis product HCl by NaHCO_3_, indicating the presence of more carboxyl groups on the membrane surface [35]. The hydrolyzed TMC provided more carboxyl groups as shown by the zeta potential, indicating that the NaHCO_3_ addition resulted in more carboxyl groups on the surface, effectively promoting the extent of the reaction. Adding NaHCO_3_ (a proton scavenger) may also alter the overall IP kinetics and, thus, the repulsive film’s thickness, surface area, and cross-linking [28]. The polymerization reaction of MPD and TMC was effectively promoted, which led to a decrease in the reverse salt flux and an increase in the salt interception capacity of the TFC-FO membranes. In Figure 8, it can be seen that the Js/Jw value showed a decreasing trend with the increase in sodium bicarbonate concentration, indicating that adding sodium bicarbonate effectively improved the performance of the TFC-FO membranes to some extent.

## 4. Conclusions

In this study, the micro-nano structure of the PA layer was adjusted by adding sodium bicarbonate to the aqueous phase, systematically demonstrating changes in its roughness with the addition of sodium bicarbonate. The experiments showed a systematic transition from the classical leaf nodule morphology by simply adding NaHCO_3_ (up to a concentration of 8 wt%). The effect of the change in roughness on the water flux was explored. As the roughness increased, leading to an increase in the membrane area of the polyamide layer, more concentration polarization occurred, and the water flux decreased. These nano-foam features significantly reduced the water flux of the forward-osmosis membrane. Adding NaHCO_3_ (a proton scavenger) could also change the overall IP kinetics and thus the degree of polymerization of the PA layer. The polymerization reaction of MPD and TMC was effectively promoted, which led to the reduction in the reverse salt flux and the improvement of the salt interception capacity of the TFC-FO membranes, thus improving their comprehensive performance.

## Figures and Tables

**Table 1:** Base film pore size and back opening.

Name	Number Density (Counts/μm^2^)	Diameter, dp (nm)
pores on substrate	349 ± 26	14.71 ± 0.21
TFC-0	22 ± 3	63.35 ± 12.4
TFC-2	32 ± 4	88.31 ± 16.0
TFC-4	73 ± 2.5	55.45 ± 10.5
TFC-6	92 ± 3	64.86 ± 11.8
TFC-8	94 ± 3.5	65.81 ± 9.5

## Data Availability

No new data created.
